# Transitions in the Cognitive Frailty States in Community-Living Older Adults: A 6-Year Prospective Cohort Study

**DOI:** 10.3389/fnagi.2021.774268

**Published:** 2021-12-01

**Authors:** Huiyu Tang, Huan Zhu, Qianqian Sun, Hai Qin, Shuang Wang

**Affiliations:** ^1^The Center of Gerontology and Geriatrics and National Clinical Research Center for Geriatrics, West China Hospital, Sichuan University, Chengdu, China; ^2^Internal Medicine Department, Pingyi Community Health Service Center, Dujiangyan, China

**Keywords:** community-living, elderly, cognitive frailty (CF), transitions, risk factors

## Abstract

**Background:** Frailty is a multidimensional concept, including physical, cognitive, social, sensorial, psychological, and nutritional phenotypes. Among these phenotypes, cognitive frailty is the most widely investigated, which is related to many adverse health outcomes in older individuals. Whether cognitive frailty is dynamic or how these frail phenotypes interact remains an open issue. We studied the rate of these changes over time and their associated factors in a 6-year follow-up cohort.

**Methods:** A total of 426 Chinese community-living older adults in Dujiangyan aged 65 years or older were involved and followed up in three visits 6 years apart. Frailty and cognitive function were assessed using the FRAIL scale and the Mini-Mental State Examination scale. Demographic information, geriatric syndrome, and social interaction status were studied. Rates of transitions in cognitive frailty states and associated risk factors were studied. We used the stepwise logistic regression model to analyze risk factors.

**Results:** At baseline, 18.8% of participants were only in the physical frailty (PF) or mild cognitive impairment (MCI) group, and 0.09% of participants were in the cognitive frailty group. By the end of 6 years, 62 (14.5%) participants had died, and the rates of only PF or MCI group and cognitive frailty group increased to 36.2 and 3.3%, respectively. Also, 199 (46.7%) participants had deteriorated compared with the baseline. The multivariate regression analysis showed that older (OR = 1.12, 95% CI = 1.07 − 1.16, *P* < 0.001), smoker (OR = 2.15, 95% CI = 1.37 − 3.39, *P* = 0.001), poor self-evaluation health status (OR = 1.93, 95% CI = 1.06 − 3.51, *P* = 0.033), and malnutrition (OR = 2.07, 95% CI = 1.21 − 3.52, *P* = 0.008) were risk factors for worsening, whereas willing to make new friends (OR = 0.61, 95% CI = 0.38 − 0.96, *P* = 0.032) was associated with 39% lower chance of deterioration.

**Conclusion:** Cognitive frailty is a dynamically changing state, where transitions may be influenced by multidimensions. Multidimensional monitoring of a wide range of events occurring in aging may be the best way to act early. We hope our study may serve as a starting point for redefining the definition of cognitive frailty by covering different frailty domains.

## Introduction

The latest global metrics on life expectancy showed that the populations of older ages are increasing substantially GBD 2017 DALYs and HALE Collaborators (2018). It is predicted that the number of people who aged ≥ 65 years may reach up to 1.5 billion in 2050 (Zupo et al., 2020). In older age, multiple subclinical and age-related comorbidities and the occurrence of stressors may exacerbate the functional decline in the physiological reserves of several systems, which would then result in a homeostatic imbalance or frailty (Panza et al., 2018). Frailty is a multidimensional geriatric syndrome characterized by increased vulnerability to stressors as a result of the reduced capacity of different physiological systems (Kelaiditi et al., 2013; Morley et al., 2013; Cesari et al., 2017). It is associated with increased risk of adverse health-related outcomes including falls, disability, hospitalizations, and mortality (Fried et al., 2004; Abellan van Kan et al., 2008; Graham et al., 2009; Panza et al., 2011; Castellana et al., 2021). At present, even though the underlying mechanisms of frailty are not fully understood, this heterogeneous clinical syndrome is known to be not only a physical or biological dimension but also a multidimensional concept, including physical, cognitive, social, sensorial, psychological, and nutritional phenotypes (Panza et al., 2018). Based on different pathogeneses, frailty can be divided into physical frailty, cognitive frailty, psychosocial frailty, and nutritional frailty. Psychosocial frailty is defined as a state of being at risk of losing social and general resources, activities, or abilities that are important for fulfilling one or more basic social needs during the lifespan (Bunt et al., 2017). A growing number of evidence suggests that social frailty may be a predictor of adverse outcomes, such as mortality (Garre-Olmo et al., 2013), disability (Teo et al., 2017; Tsutsumimoto et al., 2017), and cognitive outcomes (Tsutsumimoto et al., 2017). Nutritional frailty is a less explored concept, while it was first defined as a state commonly seen in vulnerable older adults, characterized by sudden, significant weight loss and loss of muscle mass and strength, or an essential loss of physiological reserves, making the individual susceptible to disability, in 2002 (Bales and Ritchie, 2002). However, as yet, no operational definition for this phenotype has been proposed (Kinney, 2004). In a population-based study, subjects with nutritional frailty were at higher risk for all-cause mortality than those with physical frailty (Zupo et al., 2021). Among these phenotypes, cognitive frailty is the most widely investigated phenotype, and it is increasingly acknowledged as a fundamental determinant of the vulnerability and resilience of an individual to stressors (Canevelli et al., 2014). In 2013, the International Academy on Nutrition and Aging (IANA) and the International Association of Gerontology and Geriatrics (IAGG) consensus panel (Kelaiditi et al., 2013) proposed the operational definition of cognitive frailty characterized by the simultaneous presence of both physical frailty and cognitive impairment [clinical dementia rating (CDR) = 0.5], and exclusion of concurrent AD dementia or other dementias. Physical frailty (PF) and cognitive impairment have similar time trajectories and pathological mechanisms, and they interact with each other (Furtado et al., 2019). A number of cross-sectional or longitudinal studies have shown that cumulative negative effects are often detected when they coexist, significantly increasing all-cause mortality or other adverse outcomes (Roppolo et al., 2017; St John et al., 2017). Previous studies indicate that frailty (Lang et al., 2009; Lee et al., 2014; Fan et al., 2019) or cognition (DeCarli, 2003) has been recognized to be a dynamic state over time, and not all will decline. However, whether cognitive frailty is dynamic or how these frail phenotypes interact remains an open issue. Understanding who is prone to decline and who may keep stable or even go back to healthy will allow clinicians to focus on people at the highest risk for early interventions (Lee et al., 2014). Based on the above research, we tried to study the natural transitions of cognitive frailty in a cohort of community-living older adults in Dujiangyan. We also analyzed the risk factors that may be significantly associated with the transitions, which may provide epidemiological evidence for preventive strategies.

## Materials and Methods

### Study Population

**FIGURE 1:**
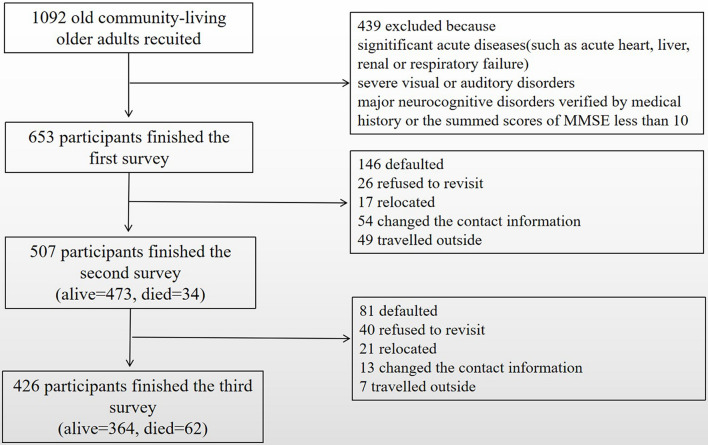
Flowchart of the study.

The data were collected from the Survey on the Disease, Psychology and Social Support of the Elderly Community-Dwelling Population in Dujiangyan, which was a prospective cohort study. We collected data using several validated scales of questionnaires, the details regarding the study design have been reported earlier (Fan et al., 2018). Included subjects were aged 65 years or older living in the Dujiangyan community region. Participants were excluded if they (1) had any significant acute disease, such as acute heart, liver, renal, or respiratory failure; (2) had severe visual or auditory disorders; and (3) were diagnosed with major neurocognitive disorders or the summed scores of MMSE <10. Figure 1 shows the flow chart of the study. The cohort was established in January 2014 and a total of 653 community-living older adults completed the first survey. The second visit and assessment were conducted in January 2017, during which 507 participants completed. The third visit and assessment were conducted in January 2020, during which 426 participants completed. The study was approved by the Ethics Review Committee of Sichuan University, and all participants signed written informed consent.

### Physical Frailty and Cognitive Function Assessment

Physical frailty at baseline and follow-up visits was evaluated using the FRAIL scale (Lopez et al., 2012) rather than Fried phenotype due to inconvenience and infeasibility of assessing grip strength and gait speed in such older community adults. The FRAIL scale contains the following aspects: fatigue, resistance, ambulation, illnesses, and unintentional weight loss. Fatigue was assessed by asking, “How often have you felt fatigued during the past 1 month?” Responses, such as “all of the time” or “most of the time,” were scored 1 point. Resistance was assessed by asking, “Did you have any difficulty in going up one floor without rest or help?” Ambulation was measured by asking, “Did you have any difficulty in walking 200 m alone without rest or help?” Illnesses were defined as the copresence of five or more chronic diseases among the following pathological conditions: hypertension, diabetes, cancer, chronic obstructive pulmonary disease (COPD), heart attack, congestive heart failure, angina, asthma, arthritis, and kidney disease. Unintentional weight loss was determined by asking, “Did you experience a weight loss of more than 5% or 4.5 kg within the past 1 year?” Each item was scored 1 point, and participants were categorized into three groups, namely, robust (score = 0), pre-frail (score = 1–2), and frail (score = 3–5), based on the summed score.

Ruan et al. (2015) divided cognitive frailty into two subtypes: reversible cognitive frailty and potential reversible cognitive frailty. Reversible cognitive frailty indicates PF and subjective cognitive decline (SCC), which may occur at the pre-mild cognitive impairment (pre-MCI) stage. Potentially reversible cognitive frailty is MCI (CDR = 0.5), which had been discussed in detail by the consensus group. In this study, Mini-Mental State Examination (MMSE) scale was used to diagnose cognitive impairment that primarily adapted to study potential reversible cognitive frailty, while less attention was paid to recognizing reversible cognitive frailty (Bu et al., 2021). Based on their educational level, the cutoff of MCI in this study was adjusted as follows: for participants who were illiterate, who had a primary school, or who had a secondary school and above, the cutoff values were ≤ 17, ≤ 20, and<24, respectively.

### The Definition of Transitions in Cognitive Frailty

According to the presence of frail and/or cognitive impairment, participants were classified into four groups, namely, (1) normal: no evidence of PF or MCI; (2) only PF or MCI: physically frail older adults with normal cognition or adults with no PF but already exhibiting MCI; (3) cognitive frailty: with the simultaneous presence of PF or MCI; and (4) death. The severity of all the four groups is progressive.

### Physical Function, Mortality, and Other Variables

Physical function was evaluated using the Instrumental Activity of Daily Living (IADL) Scale including the following items: shopping, cooking, doing housework, financial management, making telephone calls, and taking medication (Graf, 2008). Disability was defined as requiring assistance on one or more IADL item(s). The mortality data were collected from local government records or self-reports of family members. We also collected demographic information, including age, gender, education, marital status, smoking and alcohol drinking habits, self-evaluation health status, medical insurance, and social interaction status. Education was classified as illiterate, primary school, or secondary school and above. Marital status was divided into with or without a mate. Smoking habits were categorized as never smoked and smoker. Alcohol drinking habits were categorized as never drank and drinker. Self-evaluation health status was divided into five levels, namely, excellent, good, vulnerable, bad, and very bad, and the last two levels were defined as poor self-evaluation health status. Social interaction status was assessed by asking the individuals if they were willing to participate in community activities or make new friends. In addition, we assessed several geriatric syndromes. Depression was assessed using the Geriatric Depression Scale (GDS-15), with a score of 5 or above. Nutritional status was evaluated using the Mini-Nutrition Assessment Scale (MNA-SF), with a score of 12 or less considered to indicate malnutrition (Rubenstein et al., 2001). Chronic pain was defined as a continuous pain for more than 1 month. Visual impairment was assessed by self-report of vision loss or needing to wear glasses.

### Statistical Analysis

Statistical analyses were performed using SPSS Statistics (version 25.0) and EpiData (version 3.1). In this study, data were expressed using either mean ± standard deviation (X ± SD) and median (p25 − p75) for continuous variables or frequencies and percentages for categorical variables. The differences between participants were tested using either unpaired Student’s *t*-test or Wilcoxon rank-sum (Mann-Whitney) test for continuous variables, and the chi-square test for categorical variables. We used logistic regression analysis to identify factors associated with the transitions in cognitive frailty and then confirmed their independent contributions with multiple stepwise logistic regression analyses. A two-sided *P* value of 0.05 was considered statistically significant.

## Results

### The Overall Characteristics of Participants at Each Visit

Table 1 summarizes the overall characteristics of the study population. The whole sample (*n* = 653) was dominated by female participants (61.3% female participants vs. 28.7% male participants) at baseline. The age range was 72.0 (67.0, 77.0), and the percentage of illiteracy was 20.2%. The prevalence of PF, MCI, and cognitive frailty was 6.1, 16.8, and 1.7% at baseline, respectively. In the second survey, we found a higher proportion of poor self-evaluation health status (*P* = 0.043), malnutrition (*P* < 0.001), and IADL disability (*P* < 0.001). In the third survey, MCI and cognitive frailty participants showed a growing prevalence with age (*P* < 0.001, *P* = 0.007). We also found a higher proportion of smoking (*P* < 0.001) and depression (*P* < 0.001) based on the second follow-up, but less interested in participating in community activities (*P* < 0.001) or making new friends (*P* = 0.004).

**TABLE 1:** The overall characteristics of participants at each visit.

	**First survey**	**Second survey**	**Third survey**
	**alive (*n* = 653)**	**alive (*n* = 473)**	**alive (*n* = 364)**
Status (Dead) (%)	–	34 (6.7)	62 (14.5)
**Demographic**			
Age (year)	72.0 (67.0, 77.0)	74.0 (70.0, 79.0)	77.0 (72.0, 82.0)
Female (%)	400 (61.3)	283 (59.8)	219 (60.2)
Illiteracy (%)	132 (20.2)	90 (19.0)	70 (19.2)
Having a mate (%)	453 (69.4)	330 (69.8)	209 (57.4)
Smoking (%)	232 (35.5)	177 (37.4)	178 (48.9)
Drinking (%)	218 (33.4)	186 (39.3)	130 (35.7)
Having medical insurance (%)	606 (92.8)	456 (96.4)	344 (94.5)
Poor self-evaluation health status (%)	107 (16.4)	89 (18.8)	81 (22.3)
**Geriatric syndrome**			
Depression (%)	16 (2.5)	19 (4.0)	39 (10.7)
Malnutrition (%)	170 (26.0)	190 (40.2)	101 (27.7)
Chronic pain (%)	363 (55.6)	264 (55.8)	210 (57.7)
Visual impairment (%)	442 (67.7)	312 (66.0)	257 (70.6)
Physical frailty (%)	40 (6.1)	34 (7.2)	21 (5.8)
Mild cognitive impairment (%)	110 (16.8)	61 (12.9)	161 (44.2)
Cognitive frailty (%)	11 (1.7)	7 (1.5)	14 (3.8)
IADL disability (%)	22 (3.4)	56 (11.8)	77 (21.2)
**Social interaction status**			
Participating in community activities (%)	302 (46.2)	268 (56.7)	91 (25.0)
Making new friends (%)	429 (65.7)	311 (65.8)	208 (57.1)

*IADL, instrumental activity of daily living. The differences between participants were tested using either unpaired Student’s *t*-test or Wilcoxon rank-sum (Mann-Whitney) test for continuous variables, and chi-square test for categorical variables.*

### Characteristics of Participants Who Did or Did Not Complete All Visits

By the end of 6 years, a total of 426 Chinese community-dwelling participants completed all three visits. Table 2 compares the characteristics of participants who did or did not complete all visits. Those who did not return for the follow-up visits were older and had a higher proportions of women, malnutrition, and chronic pain at baseline. Other variables about demographic information, geriatric syndrome, and social interaction status were comparable between the two groups.

**TABLE 2:** Characteristics of participants who did or did not complete three visits.

	**Did not complete**	**Completed**	***P***
		
	***n = 227***	***n = 426***	
**Demographic**			
Age (year)	73.0 (69.0, 78.0)	71 (67.0, 76.0)	0.005*
Female (%)	151 (66.5)	249 (58.5)	0.044*
Illiteracy (%)	50 (22.0)	82 (19.2)	0.400
Having a mate (%)	151 (66.5)	302 (70.9)	0.248
Smoking (%)	70 (30.8)	162 (38.0)	0.067
Drinking (%)	72 (31.7)	146 (34.3)	0.510
Having medical insurance (%)	210 (92.5)	396 (93.0)	0.833
Poor self-evaluation health status (%)	35 (15.4)	72 (16.9)	0.626
**Geriatric syndrome**			
Depression (%)	4 (1.8)	12 (2.8)	0.406
Malnutrition (%)	70 (30.8)	100 (23.5)	0.041*
Chronic pain (%)	114 (50.2)	249 (58.5)	0.044*
Visual impairment (%)	152 (67.0)	290 (68.1)	0.772
Physical frailty (%)	16 (7.0)	24 (5.6)	0.473
Mild cognitive impairment (%)	46 (20.3)	64 (15.0)	0.088
IADL disability (%)	11 (4.8)	11 (2.6)	0.127
**Social interaction status**			
Participating in community activities (%)	103 (45.4)	199 (46.7)	0.744
Making new friends (%)	149 (65.6)	280 (65.7)	0.982

The differences between participants were tested using either unpaired Student’s *t*-test or Wilcoxon rank-sum (Mann-Whitney) test for continuous variables, and chi-square test for categorical variables, *P < 0.05.

### Status at Follow-Up Visits

We investigated the changes in the number of different cognitive frailty states over time. Table 3 shows the rates of different states in three visits. In the second survey, the number of normal people significantly decreased (−6.8%, *P* = 0.018) and the number of people who had died significantly increased (+8.0%, *P* < 0.001) compared with baseline, while the rates of other two states were comparable to the first survey. In the third survey, the number of normal people further decreased (−34.3%, *P* < 0.001), and the number of people who had died further increased (+14.5%, *P* < 0.001). Of note, we observed a significant increase in only PF or MCI state and cognitive frailty state after 6 years. Therefore, we speculate that it will take a relatively long time for transitions from normal to different cognitive frailty states.

**TABLE 3:** The rates of different cognitive frailty states in three visits.

**Cognitive frailty states**	**First survey**	**Second survey**		**Third survey**	
	***n* = 426**	***n* = 426**	***P***	***n* = 426**	***P***
Normal	342 (80.3)	313 (73.5)	0.018*	196 (46.0)	<0.001^#^
Only PF or MCI	80 (18.8)	73 (17.1)	0.532	154 (36.2)	<0.001^#^
Cognitive frailty	4 (0.9)	6 (1.4)	0.525	14 (3.3)	0.017^#^
Death	0 (0.0)	34 (8.0)	<0.001*	62 (14.5)	<0.001^#^

PF, physical frailty; MCI, mild cognitive impairment. *P < 0.05, first survey vs. second survey; ^#^*P* < 0.05, first survey vs. third survey.

Table 4 shows the changes in status between baseline and follow-up visits. At baseline, 18.8% of participants were only in the PF or MCI group, and 0.09% of participants were in the cognitive frailty group. By the end of 3 years, 34 (8.0%) participants had died, and mortality increased significantly with increasing cognitive frailty at baseline (*P* = 0.011). By the end of 6 years, 199 (46.7%) participants had remained stable, 199 (46.7%) participants had deteriorated, and 28 (6.6%) participants had improved. In the third survey, nearly half of the people in the normal group at baseline had stayed in the same state, whereas more than one-third had worsened into the only PF or MCI group, and a small part had degenerated into cognitive frailty or died. Among the only PF or MCI at baseline, 31.3% of participants recovered into the normal state, whereas one-third worsened into cognitive frailty or died. Among the cognitive frailty, 25% of participants recovered to the normal state, and 50% of participants recovered to only PF or MCI state, but one-fourth of participants had died.

**TABLE 4:** Transitions in cognitive frailty states in community-living older adults in three visits 6 years apart.

**First survey**	**Second survey**				**Third survey**				**Total**
	**Normal**	**Only PF or MCI**	**Cognitive frailty**	**Death**	**Normal**	**Only PF or MCI**	**Cognitive frailty**	**Death**	
Normal	261 (76.3)	59 (17.3)	1 (0.3)	21 (6.1)	170 (49.7)	123 (36.0)	9 (2.6)	40 (11.7)	342
Only PF or MCI	50 (62.5)	13 (16.3)	5 (6.2)	12 (15.0)	25 (31.3)	29 (36.2)	5 (6.2)	21 (26.3)	80
Cognitive frailty	3 (75.0)	0 (0.0)	0 (0.0)	1 (25.0)	1 (25.0)	2 (50.0)	0 (0.0)	1 (25.0)	4

*Data outside the brackets were frequencies and inside were percentages.*

### Multivariate Models

Table 3 indicates that cognitive frailty states obviously changed in the third survey, so we compared the first survey with the third survey to analyze the related factors. Table 5 shows the multiple stepwise logistic regression models of factors significantly associated with transitions in cognitive frailty over 6 years. Univariate analysis indicated that age, gender, marital status, smoking habit, self-evaluation health status, nutritional status, and social interaction status may influence the changes. After adjusting for age, gender, smoking, self-rated health, nutritional status, and social interaction status, the remaining significant factors were older (OR = 1.12, 95% CI = 1.07 − 1.16, *P* < 0.001), smoker (OR = 2.15, 95% CI = 1.37 − 3.39, *P* = 0.001), poor self-evaluation health status (OR = 1.93, 95% CI = 1.06 − 3.51, *P* = 0.033), malnutrition (OR = 2.07, 95% CI = 1.21 − 3.52, *P* = 0.008), and willing to make new friends (OR = 0.61, 95% CI = 0.38 − 0.96, *P* = 0.032).

**TABLE 5:** Multiple stepwise logistic regressions: factors significantly associated with transitions in cognitive frailty over 6 years.

	**Univariate analysis**		**Multivariate stepwise analysis**	
	**OR(95%**CI**)**	**P**	**O(95%**CI**)**	**P**
Age (year)	1.13 (1.09, 1.17)	<0.001*	1.12 (1.07, 1.16)	<0.001^#^
Female (%)	0.52 (0.35, 0.78)	0.002*		
Illiteracy (%)	1.26 (0.76, 2.09)	0.370		
Having a mate (%)	0.68 (0.44, 1.05)	0.078		
Smoking (%)	2.15 (1.42, 3.26)	<0.001*	2.15 (1.37, 3.39)	0.001^#^
Drinking (%)	1.34 (0.88, 2.04)	0.168		
Medical insurance (%)	1.65 (0.73, 3.74)	0.228		
Poor self-evaluation health status (%)	2.05 (1.18, 3.56)	0.011*	1.93 (1.06, 3.51)	0.032^#^
Depression (%)	1.00 (0.25, 4.06)	1.000		
Malnutrition (%)	2.55 (1.56, 4.16)	<0.001*	2.07 (1.21, 3.52)	0.008^#^
Chronic pain (%)	1.02 (0.69, 1.52)	0.919		
Visual impairment (%)	0.91 (0.60, 1.39)	0.670		
Physical frailty (%)	3.46 (0.94, 12.76)	0.063		
Mild cognitive impairment (%)	0.66 (0.35, 1.25)	0.203		
IADL disability (%)	2.38 (0.60, 9.34)	0.213		
Participating in community activities (%)	0.65 (0.44, 0.97)	0.035*		
Making new friends (%)	0.52 (0.34, 0.80)	0.002*	0.61 (0.38, 0.96)	0.032^#^

We used the stepwise logistic regression model to analyze the related risk factors. *P < 0.05, unadjusted; ^#^*P* < 0.05, adjusted age, gender, smoking, self-rated health, nutritional status, and social interaction status.

## Discussion

This is the first study to report cognitive frailty transitions among Chinese community-living older adults. The overall prevalence of cognitive frailty in this older population was 1.7%, and it significantly increased after 6 years (to 3.8%). There was a higher proportion of smoking, poor self-evaluation health status, depression, malnutrition, and IADL disability with aging. Meanwhile, we found a majority of participants remained stable or had deteriorated after 6 years, but about 6.6% of participants reverted to a better state. Among the participants who had improvement, 89.3% of the individuals were from the only PF or MCI group at baseline, indicating interventions should focus on the phase of precognitive frailty. Among the participants who became worse, 86.4% of the individuals were from the normal group at baseline, suggesting prevention should focus on healthy elders.

The reported prevalence of cognitive frailty ranges from 0.9 to 40.0%, according to different operational definitions and study samples (Canevelli and Cesari, 2017). The highest prevalence of cognitive frailty was observed in patients with advanced heart failure referred for heart transplantation (40.0%) (Jha et al., 2016). In community-based studies, the prevalence of cognitive frailty is markedly lower (0.9–1.8%), which is consistent with our result. From the standpoint of the link between frailty and aging, a review of previous studies demonstrated that aging is the strongest risk factor for many chronic medical conditions. Such age-related susceptibility is thought to be caused by a progressive imbalance between the challenges associated with internal and external stressors (i.e., sensory impairment, psychosocial stress, diseases, and injuries) and progressively failing resilience mechanisms, eventually leading to break in the physiological homeostasis that is clinically manifested as frailty, disability, and death (Panza et al., 2018). In our study, the prevalence of cognitive frailty increased significantly with aging, and older age was a risk factor of worsening, which confirmed the finding (Panza et al., 2015) that cognitive frailty is an age-related disease. Smoking is an important modifiable lifestyle factor, and there have been some studies focusing on the relationship between smoking and frailty or cognition. Kojima et al. (2015) reported that smoking could be used as a predictor of worsening frailty status in the community-dwelling population. Wei et al. (2020) showed that smoking patients with chronic schizophrenia exhibited more severe cognitive impairment than non-smoking patients, especially in working memory and executive function. In this study, we found that smoking was not only a risk factor for worsening but also the strongest independent risk factor. Self-rated health is known as a rating of the overall health status of an individual which is driven by cognitive and psychological processes in a previous study (Jylhä, 2009). Myers et al. (2014) found that poor self-rated health was a factor associated with the risk of frailty development in myocardial infarction patients. In our study, we confirmed that it significantly increased the likelihood of worsening cognitive frailty. Depression and frailty are important issues affecting older adults as they share several clinical characteristics such as reduced interest, poor sleep, and loss of energy. Previous study found that the diabetic elderly with depression symptoms are more likely to suffer from cognitive frailty (Linglin et al., 2020). Although there is no relationship between depression and cognitive frailty, we observed the prevalence of depression increased significantly with aging. Nutrition is a key element in most frailty concepts, both energy and protein intake are major determinants of nutritional status (Zupo et al., 2020). Undernutrition rather than overnutrition is the main cause for concern in aging since its relation to morbidity is stronger than that of obesity (Stratton et al., 2004), as muscle loss together with weakness and age-related impaired muscle function leads to frailty, sarcopenia, and disability (Cruz-Jentoft et al., 2017). Older populations are less inclined to consume protein due to a decrease in appetite and food intake, leading to a higher risk of malnutrition. In our study, participants who were malnourished increased the risk of worsening cognitive frailty by almost two times. Therefore, nutrition may be a targetable intervention with the potential to modulate the frailty risk. In addition, Roberts et al. (2012) suggested that an adequate balance of dietary intake can help maintain optimal cognitive function in the elderly. It has been shown that frailty is associated with low levels of well-being and life satisfaction. Berglund et al. (2016) found that social contacts could increase the likelihood of being satisfied with life (OR = 2.44, 95% CI = 1.24 − 4.80, *P* = 0.01), and the result was consistent with another research (Duppen et al., 2019). We confirmed this conclusion in our study. We found that participants who were willing to make new friends can significantly reduce the chance of worsening.

**FIGURE 2:**
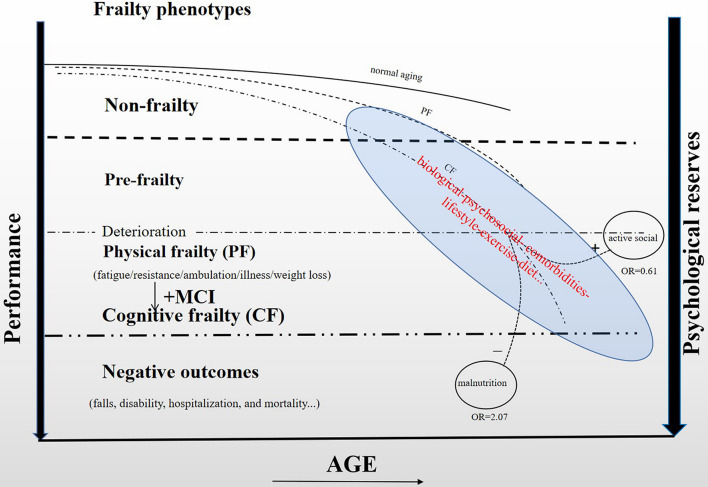
Development of different frailty phenotypes with aging.

As a crucial general concept for prevention, frailty is currently considered as “primary” or “preclinical” when the state is not associated directly with a specific disease or when there is no substantial disability (Boockvar and Meier, 2006). In this context, frailty seems more appropriate to be a unidimensional/physical phenotype (Fried et al., 2001). In contrast, frailty is considered “secondary” or “clinical” when it is associated with comorbidity and/or disability (Strandberg and Pitkälä, 2007). It appears to be better defined with the model linking frailty to the accumulation of deficits (Mitnitski et al., 2001; Rockwood et al., 2005). In this context, frailty is multidimensional sharing many similar associations with sociodemographic, lifestyle, health status, and behavioral profiles. For example, evidence from the Singapore Longitudinal Aging Study (SLAS) indicated that participants with PF, mental frailty, and/or social frailty were more likely to be older, women, single, smoking, malnutrition, underweight, and to have medical morbidity (Teo et al., 2019). In our study, we also analyzed the worsening factors from these dimensions, and the conclusion was similar to SLAS. As aging is a non-modifiable risk factor, the control of potentially modifiable factors, such as exercise and diet, lifestyle (e.g., smoking or drinking habit), psychiatric comorbidities (e.g., depression), biological (e.g., metabolic deficits), or psychosocial factors (e.g., social isolation), maybe the key to reverse the progress of frailty (Mantovani et al., 2020). We hypothesize that the development of different frailty phenotypes with aging as shown in Figure 2 after summarizing our results. Inside the blue circle are a few potentially modifiable factors that may have an impact on the trajectories of frailty. However, the impact of different factors at different stages of life is still unclear, and it deserves future studying. In any case, a bio-psycho-social model approach to frailty may be more comprehensive and then contribute to delaying the occurrence of adverse health-related outcomes.

## Limitations

This study is not without limitations. First, our cohort is not randomly selected; there may be selection bias. But it was likely a representative sample of community-living elders, as the prevalence of cognitive frailty is consistent with previous studies. Second, the loss ratio of follow-up was more than 20% because of long-time follow-up, which may reduce the efficiency of statistical analysis. But we had compared the characteristics of participants who did or did not complete all visits, finding that most of the variables were comparable. Therefore, our conclusion may also apply to participants who lost to follow-up. Finally, there might have been other potential risk factors of cognitive frailty in the study, such as neuropsychiatric disease (i.e., delirium, Parkinson’s disease, and transient ischemic attack), which may affect cognition.

## Conclusion and Future Directions

The research on frailty is ongoing, but no consensus on its definition has yet been reached and lack of uniform assessment instruments. Whether older people with PF are more at risk for developing adverse outcomes if they also suffer from cognitive, social, psychological, and nutritional frailty needs further exploration. In our study, cognitive frailty is a dynamically changing state, in which transitions may be influenced by multidimensions. Older age, smoking, poor self-evaluation health status, and malnutrition were associated with worsening, but willing to make new friends was a protective factor. In other words, different frailty phenotypes may interact with each other, even though the underlying mechanism is not clear now. Therefore, multidimensional monitoring of a wide range of events occurring in aging may be the best way to act early. However, from the perspective of health economics, it is not cost-effective to simultaneously evaluate a series of complicated frailty scales among community-living older adults. As cognitive frailty is a fundamental determinant of the vulnerability of an individual to stressors, it is more economical and effective for screening targeting on it. We hope our study may serve as a starting point for redefining the definition of cognitive frailty by covering different frailty domains. Also, we hope to construct a cognitive frailty predictive model by analyzing the results in the future.

## Data Availability Statement

The raw data supporting the conclusions of this article will be made available by the authors, without undue reservation.

## Ethics Statement

The studies involving human participants were reviewed and approved by the Ethics Review Committee of Sichuan University. The patients/participants provided their written informed consent to participate in this study.

## Author Contributions

HT assisted with study design, data analysis, and manuscript writing. HZ and QS helped with the interpretation of results. HQ helped in collecting data. SW led the development of the study concept, secured funding for data collection, and provided critical comments. All authors read and approved the final manuscript.

## Conflict of Interest

The authors declare that the research was conducted in the absence of any commercial or financial relationships that could be construed as a potential conflict of interest.

## Publisher’s Note

All claims expressed in this article are solely those of the authors and do not necessarily represent those of their affiliated organizations, or those of the publisher, the editors and the reviewers. Any product that may be evaluated in this article, or claim that may be made by its manufacturer, is not guaranteed or endorsed by the publisher.
